# Characterisation of fungal contamination sources for use in quality management of cheese production farms in Korea

**DOI:** 10.5713/ajas.19.0553

**Published:** 2019-10-21

**Authors:** Sujatha Kandasamy, Won Seo Park, Jayeon Yoo, Jeonghee Yun, Han Byul Kang, Kuk-Hwan Seol, Mi-Hwa Oh, Jun Sang Ham

**Affiliations:** 1 Animal Products Research and Development Division, National Institute of Animal Science, Rural Development Administration, Wanju 55365, Korea

**Keywords:** Fungal Diversity, Cheese Ripening Rooms, Dairy Farms, Contaminant Sources

## Abstract

**Objective:**

This study was conducted to determine the composition and diversity of the fungal flora at various control points in cheese ripening rooms of 10 dairy farms from six different provinces in the Republic of Korea.

**Methods:**

Floor, wall, cheese board, room air, cheese rind and core were sampled from cheese ripening rooms of ten different dairy farms. The molds were enumerated using YM petrifilm, while isolation was done on yeast extract glucose chloramphenicol agar plates. Morphologically distinct isolates were identified using sequencing of internal transcribed spacer region.

**Results:**

The fungal counts in 8 out of 10 dairy farms were out of acceptable range, as per hazard analysis critical control point regulation. A total of 986 fungal isolates identified and assigned to the phyla Ascomycota (14 genera) and Basidiomycota (3 genera). Of these *Penicillium*, *Aspergillus*, and *Cladosporium* were the most diverse and predominant. The cheese ripening rooms was overrepresented in 9 farms by *Penicillium* (76%), while *Aspergillus* in a single farm. Among 39 species, the prominent members were *Penicillium commune*, *P. oxalicum*, *P. echinulatum*, and *Aspergillus versicolor*. Most of the mold species detected on surfaces were the same found in the indoor air of cheese ripening rooms.

**Conclusion:**

The environment of cheese ripening rooms persuades a favourable niche for mold growth. The fungal diversity in the dairy farms were greatly influenced by several factors (exterior atmosphere, working personnel etc.,) and their proportion varied from one to another. Proper management of hygienic and production practices and air filtration system would be effective to eradicate contamination in cheese processing industries.

## INTRODUCTION

In Korea, both importation as well as domestic consumption of cheese is gradually increasing in the consumer market and food processing industry. Domestic cheese manufacturers are more focusing to develop different products leading to an increase in production [1]. Although cheese consumption is safe, still numerous outbreaks of foodborne pathogens are reported from cheese. Risk assessment of cheese spoilage by pathogenic bacteria is well-documented [2]. In contrast, despite data that several molds are involved in the spoilage of dairy products [3,4], few studies have been done on identification and assessment of molds from cheeses and/their production environment [5–7].

Cheese ripening rooms provide an excellent environment for the growth of several undesirable molds [8]. *Penicillium*, *Aspergillus*, *Cladosporium*, *Alternaria*, *Fusarium*, and *Talaromyces* are most frequently reported genera in cheese spoilage [7,9,10]. In addition, some adventitious molds can also contaminate cheese during the ripening process [11]. These contaminants predominantly originate from the environments including ambient air, equipments, handling persons, raw materials and ingredients, and surfaces [12–14]. Contamination of cheese by these molds is unfavourable to cheese quality, affecting the exterior appearance, off-flavours and production of toxic secondary metabolites [7,15]. The consumption of cheese contaminated by molds and or their toxins can pose serious foodborne illness and huge financial losses [16]. In-turn to avoid or delay mold spoilage and thereby to increase the microbial quality, safety and shelf life of cheese/other dairy products, prevention approaches together with hurdle technologies are employed by commercial dairy farms. Normally, most of the traditional cheeses are manufactured in local dairy farms under poor hygienic conditions due to lack of awareness of food safety and systematization [14,17]. Although cleaning and disinfection practices considerably lower the microbial load in the production environment, surfaces are hardly sterile. Occasional examination of the microbiological population or determination of certain microbial types is required in the dairy industry [12]. The intensity of microbial contamination along with identification of the most proliferative species is significant, as they might offer a hint of problems within the production environment besides the potential risk due to the existence of toxin producers [13,18]. In order to guarantee a safe food for consumers, the producers have to focus on the implementation of suitable production and hygiene practices, execution of hazard analysis critical control point (HACCP) system and to utilize air filtration/decontamination equipments. To employ these practices, an essential knowledge of the existence and source of the organisms within the dairy farm environment (air, equipment and surfaces) is mandatory. Therefore, we performed an in-depth study on the occurrence of molds and their diversity associated within the cheese ripening rooms (wall, floor, cheese board, air and ripened cheese) in the dairy farms of Korea, in line to assess the contamination sources and upturn the effectiveness of cleaning/disinfection methods and to control the air quality.

## MATERIALS AND METHODS

### Selection of Industrial dairy farms and sample collection

Ten dairy farms producing speciality (Gouda and Cheddar) cheeses were sampled during May 2017 to March 2018 from six different provinces of the Republic of Korea. Two farms were located in Jeonbuk (B, F), three in Jeonnam (A, D, J), two in Gyeongnam (E, G) and one from each Chungnam (C) Gyeonggi (H), and Gangwon (I). The ripened cheese types produced from farms A, E, G, H, and J was Gouda, while B, C, D, I were Cheddar and Gouda, and F was Cheddar. Samples were collected at various source points (wall, floor, cheese board, air and ripened cheese) in the cheese ripening rooms of the dairy farms. All samples were transported to the laboratory using a cooler box and analysed within 24 h of arrival.

### Sample preparation and quantitative microbiological analyses

Surface sampling was taken through swabbing an area of 10 cm^2^ from the floor, wall and cheese board using a pre-wetted (0.1% w/v peptone water) 3 M Quick swab (3M, St Paul, MN, USA). For ripened cheeses, the rind (9 cm^2^ on each face) and core (11 g) samples excised aseptically were transferred into stomacher bags containing sterile 99 mL physiological saline (pre heated at 45°C) and homogenized (250 rpm, 90 s) using a Stomacher lab blender (FR/Bag Mixer; Interscience, St. Nom, France) [19]. Both cheese and surface samples were serially diluted in peptone water and 1 mL dilution was placed onto YM petrifilm (3M, USA). The indoor air was evaluated by exposing YM petrifilm (3M, USA) for 15 min under normal air circulation in plants. In all cases, the petrifilms were incubated under dark conditions at 25°C for 5 days. The total number of viable molds on the surfaces was determined in colony-forming unit (CFU)/cm^2^ and propagules on air as CFU/plate.

### Isolation of molds from the cheese ripening room

As stated above, the sample preparation and serial dilutions were done and 1 mL dilution of each sample was plated onto the yeast extract glucose chloramphenicol (YEGC) agar medium (g/L yeast extract, 5; glucose, 20; chloramphenicol, 0.1; and agar, 15) (Sigma-Aldrich, Saint-Louis, MO, USA). The plates were incubated under dark conditions at 25°C for 5 days and based on the morphological difference (shape and colour), individual colonies were selected, restreaked and further purified on YEGC agar plates. The purified isolates grown in YEGC broth were stored at −80°C in 10% glycerol until further use.

### Identification of molds

For internal transcribed spacer (ITS)-polymerase chain reaction (PCR) based molecular identification, the genomic DNA was extracted from the fungal isolates according to Al-Samarrai and Schmid [20]. The genomic DNA region covering ITS1-5.8S-ITS2 was PCR amplified using the universal ITS1 (5′-TCC GTA GGT GAA CCT GCG G - 3′) and ITS4 (5′- TCC TCC GCT TAT TGA TAT GC -3′) primers [21] and the purified PCR products (~550 bp) were sequenced using Sanger sequencing method. The obtained sequences were searched for sequence homology against known fungal species at GenBank Database using the “basic local alignment tool” (BLAST) after editing and trimming the sequences using Bioedit sequence alignment editor.

## RESULTS AND DISCUSSION

### Enumeration of cultivable molds

Molds are the most frequent contaminants in cheese industries, which provide a suitable niche (low pH, high moisture content) for their growth [8]. Contamination of cheese by molds can occur at various stages from dairy farms to dairy processing units thereby posing a crucial problem in the production process as well as the final quality [9,12,13]. The average mold counts for various control points (floor, wall, cheese board, air and ripened cheese) in the selected 10 dairy farms were depicted in Table 1. The counts in the floor of farm A and in walls of farm A and H were found to be undetectable. In contrast, the floor of farm F was higher than the detectable levels. The floor and wall of farm D showed higher counts of 5.9×10^2^ and 1.5×10^3^ cfu/cm^2^, respectively. Improper cleaning and disinfection of surfaces favour the growth of molds in the wall, floor and ceiling under humid conditions [12,13]. Cheese boards from farms B and D showed higher counts (1.9 and 1.0×10^3^ cfu/cm^2^), while from farm J and A counts were lower (0.1 and 0.3×10^3^ cfu/cm^2^). Similarly, higher mold counts were previously reported in the Turkish white cheese vats [13]. In cheese industries, air is the major contamination source and spoilage molds are more frequently reported due to airborne fungi since their spores easily dispersed into the air inside dairy plants [10]. The room air in cheese ripening rooms of farms G-I was detected with higher counts of 4.8, 2.7, and 3.0×10^2^ cfu/plate, respectively. The microbial load that grows on the cheese surface can be complex, influenced by the ripening and preparation conditions [19,22]. The counts in cheese rind samples from farms A and B were undetectable. Although molds were detectable in other farms, a higher count (3.1×10^4^ cfu/cm^2^) was recorded with farm G. This could be due to low pH, moisture content, high salt levels and temperature that occurs during cheese ripening process favours the growth of molds [17]. The cheese core samples produced from all the ten farms were below detectable levels. In our study, we revealed that 8 out of 10 dairy farms did not comply with the HACCP standards. This study provides a more accurate quantitative portrait of the fungal microflora and depicts the composition of the cheese environment varies from one farm to another.

### Richness of mold isolates

**Figure 1:**
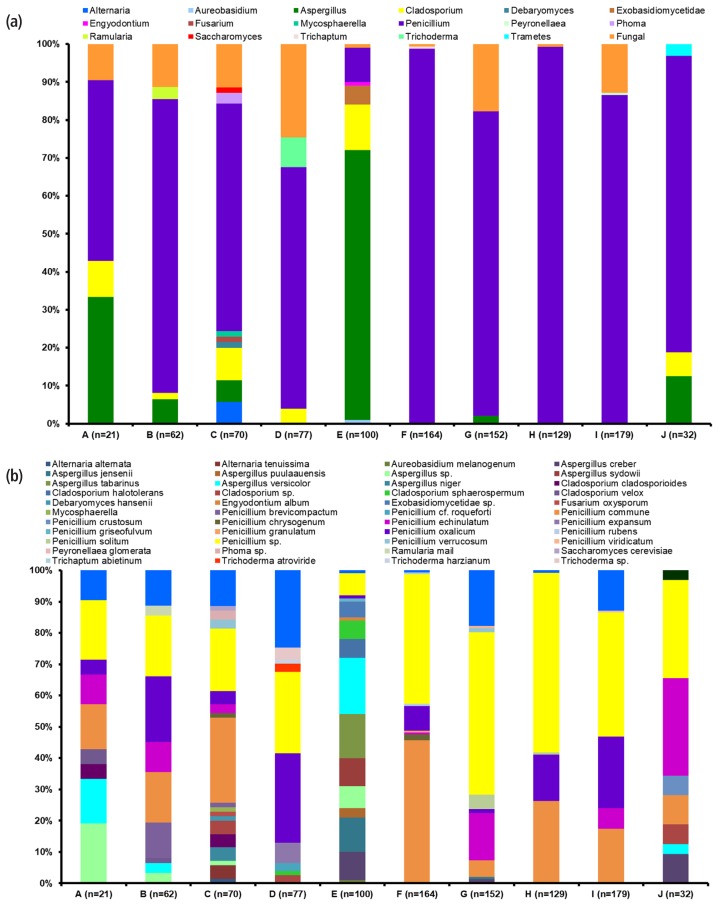
Relative distribution of molds at genera (a) and species (b) levels in the cheese ripening rooms across different dairy farms in Korea. Dairy farms located in provinces of Jeonbuk (A, B), Jeonnam (D, F, J), Gyeongnam (E, G), Chungnam (C) Gyeonggi (H) and Gangwon (I). The cheese types from farms A, E, G, H and J, Gouda; while B, C, D, I, Cheddar and Gouda; and F, Cheddar.

This part of study specified an insight into the biodiversity of fungal contaminants related to dairy products. To succeed in this goal, we employed phenotypic and genotypic tactics involving the sequencing of taxonomically related target genes to identify fungal isolates as precisely as possible. In our study, 986 fungal colonies were obtained from the samples collected in 10 dairy farms. Selection of colonies was based on differences in shape and colour. This approach increased the possibility of isolating a greater number of species. The selected isolates (Table 2, Figure 1a) were >150 in three farms (I, F, G), >100 in two farms (E, H), >50 in three farms and <50 in two farms (A, J).

The ITS region is preferred as the best universal barcode for fungal identification [23]. The molecular identification tool using the ITS1-5.8S-ITS2 ribosomal DNA (rDNA) region revealed that out of 986 isolates (Table 2), 895 isolates could be assigned to the phylum Ascomycota (886 isolates [90%], 14 genera and 37 species) and Basidiomycota (7 isolates [0.71%], 3 genera and 2 species), while remaining 89 isolates remain unclassified. In accordance, several studies on cheeses, dairy products spoilage and production environments illustrated that major molds identified belong to the phylum Ascomycota [4,24].

Molds in the Ascomycota phylum belong to the subdivision Pezizomycotina (12 genera; 35 species) and Saccharomycotina (2 genera; 2 species). Similar to our results, Lund et al [25] described all the molds identified from cheese spoilage as belonging to subdivision Pezizomycotina. In this study, within the Pezizomycotina subdivision, the most represented class is the Eurotiomycetes (2 genera and 21 species) followed by the Dothideomycetes (7 genera and 8 species) the Sordariomycetes (3 genera and 6 species) and the Saccharomycetes (2 genera and 2 species). Among the Eurotiomycetes, the genera *Penicillium* and *Aspergillus* were most frequently associated with dairy product spoilage, cheese ripening and production environment [3,7,26]. The molds in the Basidiomycota phylum belong to the subdivision Agaricomycotina in the class Agaricomycetes (2 genera and 1 species) and Ustilagomycotina in the class Exobasiomycete (1 genus).

Of the total 17 genera isolated, the genera *Penicillium*, *Aspergillus* (Supplementary Figure S1) and *Cladosporium* were found to be frequent with a greater number of isolates (Table 2, Figure 1a). *Penicillium* was the only genus detected in all the 10 dairy farms, whereas *Aspergillus* and *Cladosporium* were prevalent in 6 farms. Out of 17 genera, 14 were acquired in only one of the farms, indicating that the later genera did not occur widely as a regular part in the cheese/dairy environments.

The most diverse and abundant isolated genus was *Penicillium* (76%) with 750 isolates and 14 species found to be overly represented in all the cheese-ripening rooms, except for farm E (Table 3, Figure 1a). More than 100 isolates of the genus detected from farms F–J, and I, while <50 from farm A–E. The least number of isolates, 9 and 10 were obtained from farm E and A, respectively. Surprisingly, farms H and F have 129 and 164 isolates, of which 128 (99%) and 162 (98.7%), respectively were ascertained to the genus *Penicillium*. This agrees with numerous earlier studies affirming that *Penicillium* spp. is the most prevalent mold in the indoor environment of many cheese factories [9,27].

The second (9.43%) most diverse and dominant genera were *Aspergillus* (93 isolates, 7 species) followed by (2.64%) by *Cladosporium* (26 isolates, 4 species). The genera *Aspergillus* (Figure 1a; Table 4) and *Cladosporium* (Figure 1a; Table 5) were identified from 6 out of 10 dairy farms, although more frequently in farm E (76%). In contrast to *Penicillium* species, most of the *Aspergillus* and *Cladosporium* species cannot grow under low temperatures conditions, as they are generally mesophilic [3]. The indoor temperature of the farm E might certainly contributed to the existence of these species and repressed the dominance of *Penicillium* as observed in other farms. Among 100 isolates from Farm E, 71% isolates from the cheese board, floor, wall, air and cheese rinds were identified as belonging to the genera *Aspergillus*. *Aspergillus* was undetectable in four farms (D, F, H, I), while the numbers were <10 in farms from A to C, G and J. The genera *Aspergillus* and *Cladosporium* were highly represented in farm E, while *Penicillium* and *Aspergillus* in A, B, and J. The genus *Penicillium* alone completely dominated in farms F–I. (Table 3–6, Figure 1a). It is commonly agreed that the fungal dynamics in the interior environment of a dairy farm is more influenced by the exterior atmosphere [10,26]. Earlier studies have revealed dominance of the genera *Penicillium*, *Aspergillus*, and *Cladosporium* in the air of cheese ripening rooms in Portugal [8]; *Penicillium* and *Cladosporium* in a Greek dairy plant [26] and *Cladosporium*, *Alternaria*, and *Penicillium* in the indoor air of Italian dairy environments [10]. These results clearly indicated that the genera *Penicillium*, *Aspergillus*, and *Cladosporium* are not uncommon and constantly prevalent in cheese production farms and other dairy products.

Among the cheese ripening rooms, farm C had 9 genera mainly comprised of *Alternaria*, *Aspergillus*, *Cladosporium*, *Fusarium*, and *Penicillium*. All these genera have been previously described as contaminants in cheese and their production environment across various countries [9,10,13]. The occurrence of these genera in an indoor environment of a cheese production farm reveals poor hygienic processes as they were commonly attributed to the outdoor air in rural areas [8,10,26].

Despite a huge number of isolates, it is possible that the mold species actually identified were only a portion of the total present. Maybe few strains can find hard to grow on a synthetic medium or exist in such low numbers that the large numbers of the other species dominated them. Nevertheless, the outline of the fungal microflora in the dairy farms affords an idea of the complexity of this ecosystem.

### Diversity of mold species

The abundance of molds in cheese ripening rooms analysed at the species level resulted in identification of 39 species (Figure 1b, Tables 3–6). Of which only one species of *Aureobasidium*, *Debaryomyces*, *Engyodontium*, *Fusarium*, *Mycosphaerella*, *Peyronellaea*, *Saccharomyces*, *Trichaptum*, and *Trametes* were isolated. Most of the molds revealed within the cheese ripening rooms comprised of general cheese contaminants along with species not frequently isolated from cheese and other dairy products. The species of *Penicillium*, *Aspergillus*, and *Cladosporium* described in our study are already described in earlier works [8–10,27] as potentially toxigenic genera and frequent contaminants in dairy production environment.

*Penicillium* spp. is adapted to the cheese manufacturing process and they are well acclimated to the cheese production environment. Moreover, several studies [3,13,28] reported *Penicillium* species as the predominant mold in spoilage of cheeses and other dairy products. Based on a recent classification system proposed by Visagie et al [29], *Penicillium* spp. isolates fit into the section Fasciculata (*P. commune*, *P. crustosum*, *P. echinulatum*, *P. solitum*, *P. verrucosum*, *P. viridicatum*), Penicillium (*P. expansum*, *P. griseofulvum*, *P. granulatum*), Chrysogena (*P. chrysogenum*, P*. rubens*), Brevicompacta (*P. brevicompactum*), and Roquefortorum (*P. roqueforti*). Most of the *Penicillium* species belongs to section Fasciculata. Of the 14 different *Penicillium* species, 6 (*P. cf.roqueforti*, *P. crustosum*, *P. griseofulvum*, *P. granulatum*, *P. rubens*, and *P. viridicatum*) were detected in only one of the farms (Table 3, Figure 1b).

Among thirty-nine different mold species identified in this study, *P. commune* (18.6%) was especially more abundant in eight out of ten farms (except farm D and E) as contaminants from the floor, wall, cheese board, air and cheese rind (Table 3, Figure 1b). Likewise, several studies [3,9,27] reported dominance of *P. commune* on cheese environments as well as dairy products. *P. commune* is one of the most common fungi in cheese spoilage and reported as the source of discolouration on cheese surfaces and production of off-flavours in cheese factories [5,6]. The dominance of this indoor environmental fungus could be attributed to its ability to survive under low oxygen concentration and its psychrotolerant nature [6]. The conditions prevailing inside the indoor environment seems to favour its existence in the cheese dairy farms. The second and third abundant species *P. oxalicum* (10.7%) and *P. echinulatum* (5.7%), respectively were isolated from nine (except farm J) and seven farms (except farm D, E & H). The major source of *P. echinulatum* isolates was found to be cheese board, since it is very well associated with wooden products and wooden shelves in the cheese production environment [28]. *P. commune* and *P. echinulatum* were observed in the cheese core samples of few farms, as both are highly related to the cheese flora [30]. Other species like *P. crustosum*, *P. solitum*, *P. verrucosum*, *P. griseofulvum*, *P. chrysogenum*, *P. brevicompactum*, and *P. roqueforti* isolates of this study were also previously reported as common contaminants in cheese production environments and dairy products [6,8,15]. Most of the above-mentioned *Penicillium* species acclimate to low temperatures [15], consequently their existence in spoilage of dairy production environment is not unexpected.

The domination by *Penicillium* species, which includes certain toxigenic species, raise safety concerns, owing to possible mycotoxins contamination in cheese rinds [7,16,18]. Mycotoxins such as citrinin and ochratoxin A produced by *Penicillium* species have been detected in cheeses. Although *P. roqueforti* is well known for cheese ripening in blue cheese, a few strains have been reported as cheese contaminants and producers of mycotoxins, like PR toxin (*P. roqueforti toxin*), roquefortines, sofumigaclavines, and mycophenolic acid [16, 18,28]. In addition, other species in our study like *P. chrysogenum*, *P. expansum*, *P. griseofulvum*, *P. granulatum* and *P. crustosum* are able to produce roquefortine C. *P. verrucosum* can produce a combination of citrinin and ochratoxin A, while *P. crustosum* produce penitrem A and roquefortine C [16,28].

Next to the three most abundant *Penicillium* species, *Aspergillus versicolor*, *A. creber*, *A. tabarinus* and *A. jensenii* dominated in the cheese ripening rooms (Table 4, Figure 1b). *A. versicolor* was especially more abundant in farm E (18 out of 24 isolates) and except one isolate all were detected in the indoor air of cheese ripening rooms. As *A. versicolor* has airborne conidia it is most often found to dominate in the entire cheese ripening rooms, although it hardly grows on cheeses. Frequent incidence of *A. versicolor* was observed in Italian Fossa cheese and its ripening environment [30]. Among the dairy farms, *Aspergillus* species was found more dominant in farm E. Except *A. niger*, all species isolated in the study were present in farm E, while the earlier was detected in farm C. Since several years, *A. versicolor* and *A. niger* have been often reported to cause thread mold spoilage in cheeses [3]. Other major species like *A. versicolor*, *A. creber*, *A. puulaauensis*, *A. jensenii* presented in our study are possible sterigmatocystin producers in cheeses [18,31].

Our study showed that fungal contaminants belonging to the class Dothideomycetes were the second largest flora. Among them, *Cladosporium* spp. are the most common airborne species which often originated from aeration structures, dust, outdoor environments or wet spots [9,10]. The *Cladosporium* species was completely absent in farms from F–J, and <10 in numbers in farms A–D and J, although a maximum of 12 isolates was obtained in farm E (Table 5). The air and cheese board having a higher probability of harbouring this genera. *Cladosporium* spp. are well documented in spoilage of different dairy products (raw milk, butter, cream, cheese and margarine) [4] and being present in cheese environments [9,10,28]. Few species of *Cladosporium* including *C. cladosporioides*, which grows well in moistened wood surfaces and conditions with varying temperatures [9], are unsafe for human health. Correspondingly, in our study we isolated *C. cladosporioides* in the cheese board from two dairy farms (A and C). The two-halotolerant *Cladosporium* species (*C. halotolerans* and *C. sphaerospermum*) (Table 5, Figure 1b) in our study have been associated with spoilage of butter [4].

The majority of the species of *Penicillium*, *Aspergillus*, and *Cladosporium* encountered in various regions of the ripening rooms also exist in the ambient air of cheese ripening rooms. The spores of filamentous fungi might be easily disseminated via airborne propagation. These results well corroborate with the findings of previous studies on dairy production environments [8,10,26].

With respect to the other minor mold species (Table 6, Figure 1b), few species are isolated only once in a single farm. Species like *Trichoderma* (3 species), *Alterneria* (2 species) and *Exobasidiomycetidae* were the representing ones. *Debaryomyces hansenii* and *Saccharomyces cerevisiae* isolated from the cheese board in a single farm each, although they are known for frequent association with the rind and core of cheese [11,22]. Species of *Exobasidiomycetidae*, M*ycosphaerella*, and *Phoma* were undetermined in our study. To our knowledge, this is the first study that depicts the fungal diversity within the cheese ripening rooms from the dairy farms of the Republic of Korea. The background microflora is highly influenced by several indoor and outdoor sources that need severe attention to prevent any hazards to human health as well as economic losses.

## CONCLUSION

This study has disclosed new relevant evidence on the diversity and conserving resistance of mold contaminants encountered at various control points in the cheese ripening rooms. Consequently, it is necessary to implement screening procedures and or practices (e.g. good production procedures, regular hygienic measures, hazard analysis and critical control point, etc.) at particular time intervals for producing safe and quality cheese products. This will be more helpful in maintaining the cheese and other dairy processing industries from any possible contamination. Overall, this information will be helpful for the dairy farms to develop innovative production processes and adequate strategies to eliminate the existence of fungi and/or limit their development in various control points, to eradicate contaminations in cheese processing industries.

## Supplementary Data



## Figures and Tables

**Table 1:** Enumeration of molds from various sources in cheese ripening rooms across different dairy farms in Korea

Isolation source	Microbial counts1) (CFU/cm^2^, CFU/plate)2)									

	A3)	B	C	D	E	F	G	H	I	J
Room floor	ND	2.3×10^1^	6.7×10^0^	5.9×10^2^	1.5×10^1^	NC	5.1×10^1^	2.6×10^1^	1.3×10^1^	0.2×10^0^
Room wall	ND	8.0×10^0^	5.3×10^0^	1.5×10^3^	0.2×10^0^	1.6×10^2^	0.7×10^0^	ND	0.1×10^0^	0.7×10^0^
Cheese board	0.3×10^0^	1.9×10^3^	1.5×10^1^	1.0×10^3^	1.0×10^1^	NC	2.6×10^2^	5.3×10^0^	3.2×10^1^	0.1×10^0^
Room air	5.8×10^1^	6.6×10^0^	1.1×10^1^	4.1×10^1^	6.6×10^0^	2.3×10^1^	4.8×10^2^	2.7×10^2^	3.0×10^2^	3.8×10^1^
Cheese-rind	ND	ND	0.3×10^0^	1.4×10^1^	0.2×10^0^	3.1×10^0^	3.1×10^4^	4.8×10^0^	4.7×10^1^	1.1×10^1^
Cheese-core	ND	ND	ND	ND	ND	ND	ND	ND	ND	ND

ND, not detectable; NC, not counted (denotes too few colonies to estimate).

1) Values are mean of three replicates of each sample.

2) Surface area, CFU/cm^2^; air borne fungus, CFU/plate.

3) Dairy farms located in provinces of Jeonbuk (B, F), Jeonnam (A, D, J), Gyeongnam (E, G), Chungnam (C) Gyeonggi (H) and Gangwon (I). The cheese types from farms A, E, G, H and J, Gouda; while B, C, D, I, Cheddar and Gouda; and F, Cheddar.

**Table 2:** Taxonomic classification of molds in the cheese ripening rooms across different dairy farms in Korea

Division	Genera	No of species	No of isolates	No of farms
Ascomycota	*Alternaria*	2	4	1
	*Aureobasidium*	1	1	1
	*Aspergillus*	7	93	6
	*Cladosporium*	4	26	6
	*Debaryomyces*	1	1	1
	*Engyodontium*	1	1	1
	*Fusarium*	1	1	1
	*Mycosphaerella*	0	1	1
	*Penicillium*	14	750	10
	*Peyronellaea*	1	1	1
	*Phoma*	1	2	1
	*Ramularia*	1	2	1
	*Saccharomyces*	1	1	1
	*Trichaptum*	1	1	1
Basidiomycota	*Exobasidiomycetidae*	0	5	1
	*Trichoderma*	2	6	1
	*Trametes*	1	1	1
	*Fungal*	-	89	-
	Total (17 genera)	39	986	

**Table 3:** Diversity of *Penicillium* species at various control sources1) in the cheese ripening rooms across different dairy farms in Korea

Genus	Species	No of isolates										

		A2)	B	C	D	E	F	G	H	I	J	Total
*Penicillium*	*brevicompactum*	-	7 (w,b,a)	1 (a)	-	-	-	-	-	-	-	8
	*cf. roqueforti*	-	-	-	2 (w,b)	-	-	-	-	-	-	2
	*commune*	3 (a)	10 (a,w,b)	19 (a,f,w,r)	-	-	75 (a,f,w,b,r,)	8 (b,r)	34 (f,b,a,r)	31 (r)	3 (a,w)	183
	*crustosum*	-	-	-	-	-	-	-	-	-	2 (a)	2
	*chrysogenum*	-	-	1 (r)	-	-	3 (w)	-	-	-		4
	*echinulatum*	2 (a)	6 (b)	2 (a)	-	-	1 (w)	23 (f,w,b,a,r)		12(r)	10 (w,b,a,r)	56
	*expansum*	-	-	-	5 (f,w,a)	-	-	-	-	-	-	5
	*griseofulvum*	-	-	-	-	1 (b)	-	-	-	-	-	1
	*granulatum*	-	-	-	-	-	1 (w)	-	-	-	-	1
	*oxalicum*	1 (a)	13 (w,b)	3 (b,a,r)	22 (w,b,a,r)	1 (f)	13 (f,w,b,a,r)	2 (f,r)	19 (f,a,r)	41 (r)	-	115
	*rubens*	-	-	-	-	-	1 (w)	-	-	-	-	1
	*solitum*	-	-	-	-	-	-	7 (f,b,a,r)	1 (a)	-	-	8
	*verrucosum*	-	-	2 (f)	-	-	-	2 (a)	-	-	-	4
	*viridicatum*	-	-	-	-	-	-	1 (a)	-	-	-	1
	sp.	4 (a)	12 (w,b)	14 (f,w,b,a,r)	20 (f,w,b,a)	7 (f,r,a)	68 (f,w,a,r)	79 (f,w,b,a,r)	74 (f,b,a,r)	71 (r)	10 (f,a)	359
	Total (14 species)	10	48	42	49	9	162	122	128	155	25	750

1) Sources: floor (f), wall (w), cheese board (b), air (a), cheese rind (r), and cheese core (c).

2) Dairy farms located in provinces of Jeonbuk (B, F), Jeonnam (A, D, J), Gyeongnam (E, G), Chungnam (C), Gyeonggi (H), and Gangwon (I). The cheese types from farms A, E, G, H and J, Gouda; while B, C, D, I, Cheddar and Gouda; and F, Cheddar.

**Table 4:** Diversity of *Aspergillus* species at various control sources1) in the cheese ripening rooms across different dairy farms in Korea

Genus	Species	No of isolates										

		A2)	B	C	D	E	F	G	H	I	J	Total
*Aspergillus*	*creber*	-	-	-	-	9 (w,b,a,r)	-	2 (a)	-	-	3(a)	14
	*jensenii*	-	-	-	-	11 (f,w,b,a,)	-	1(a)	-	-	-	12
	*puulaauensis*	-	-	-	-	3 (w,a)	-	-	-	-	-	3
	*sydowii*	-	-	-	-	9 (f,b,r)	-	-	-	-	-	9
	*tabarinus*	-	-	-	-	14 (f,b,a)	-	-	-	-	-	14
	*versicolor*	3 (a)	2 (b,a)	-	-	18 (b)	-	-	-	-	1 (a)	24
	*niger*	-	-	3 (f)	-	-	-	-	-	-	-	3
	sp.	4 (a)	2 (w,b)	1 (r)	-	7 (f,b,a)	-	-	-	-	-	14
	Total (7 species)	7	4	4	-	71	-	3	-	-	4	93

1) Sources: floor (f), wall (w), cheese board (b), air (a), cheese rind (r), and cheese core (c).

2) Dairy farms located in provinces of Jeonbuk (B, F), Jeonnam (A, D, J), Gyeongnam (E, G), Chungnam (C), Gyeonggi (H), and Gangwon (I). The cheese types from farms A, E, G, H and J, Gouda; while B, C, D, I, Cheddar and Gouda; and F, Cheddar.

**Table 5:** Diversity of *Cladosporium* species at various control sources1) in the cheese ripening rooms across different dairy farms in Korea

*Cladosporium* sp.	No of isolates										

	A2)	B	C	D	E	F	G	H	I	J	Total
*Cladosporium cladosporioides*	1 (b)	-	3 (b,r)	-	-	-	-	-	-	-	4
*Cladosporium halotolerans*	-	-	-	-	6 (f,w,b,a)	-	-	-	-	-	6
*Cladosporium sphaerospermum*	-	-	-	1 (w)	6 (f,a)	-	-	-	-	-	7
*Cladosporium velox*	1 (b)	1 (a)	-	-	-	-	-	-	-	-	2
*Cladosporium* sp.	-	-	3 (f,a)	2 (a)	-	-	-	-	-	2 (a)	7
Total (4 species)	2	1	6	3	12	-	-	-	-	2	26

1) Sources: floor (f), wall (w), cheese board (b), air (a), cheese rind (r) and cheese core (c).

2) Dairy farms located in provinces of Jeonbuk (B, F), Jeonnam (A, D, J), Gyeongnam (E, G), Chungnam (C), Gyeonggi (H), and Gangwon (I). The cheese types from farms A, E, G, H and J, Gouda; while B, C, D, I, Cheddar and Gouda; and F, Cheddar.

**Table 6:** Diversity of other known fungal species at various control sources1) in the cheese ripening rooms across different dairy farms in Korea

Genera	Species	No of isolates										

		A2)	B	C	D	E	F	G	H	I	J	Total
*Alternaria*	*A. alternata*	-	-	1 (b)	-	-	-	-	-	-	-	4
	*A. tenuissima*	-	-	3 (b,r)	-	-	-	-	-	-	-	
*Aureobasidium*	*A. melanogenum*	-	-	-	-	1 (f)	-	-	-	-	-	1
*Debaryomyces*	*D. hansenii*	-	-	1 (b)	-		-	-	-	-	-	1
*Engyodontium*	*E. album*	-	-	-	-	1 (b)	-	-	-	-	-	1
*Exobasidiomycetidae*	*Exobasidiomycetidae* sp.	-	-	-	-	5 (b,a)	-	-	-	-	-	5
*Fusarium*	*F.oxysporum*	-	-	1 (f)	-	-	-	-	-	-	-	1
*Mycosphaerella*	*Mycosphaerella* sp.	-	-	1 (r)	-	-	-	-	-	-	-	1
*Peyronellaea*	*P. glomerata*	-	-	-	-	-	-	-	-	1 (r)	-	1
*Phoma*	*phoma* sp.	-	-	2 (f)	-	-	-	-	-	-	-	2
*Ramularia*	*R. mail*		2 (w)	-	-	-	-	-	-	-	-	2
*Saccharomyces*	*S. cerevisiae*	-	-	-	-	1 (b)	-	-	-	-	-	1
*Trichaptum*	*T. abietinum*	-	-	-	-	-	1 (a)	-	-	-	-	1
*Trichoderma*	*T. atroviride*	-	-	-	2 (f,b)	-	-	-	-	-	-	6
	*T.harzianum*	-	-	-	1 (f)	-	-	-	-	-	-	
	*Trichoderma* sp.	-	-	-	3 (b)	-	-	-	-	-	-	
*Trametes*	*T.versicolor*	-	-	-	-	-	-	-	-	-	1 (b)	1
*Fungal*	*Fungal endophyte*	2 (a)	6 (w,b)	2 (a)	2 (a,r)	-	1 (b)	-	-	15 (r)	-	28
	*Fungal* sp.		1 (w)	6 (f,a,r)	17 (f,w,b,a,r)	1 (f)	-	27 (f,w,b,a,r)	1 (r)	8 (r)	-	61
	Total (15 species)	2	9	17	25	9	2	27	1	24	1	117

1) Sources: floor (f), wall (w), cheese board (b), air (a), cheese rind (r), and cheese core (c).

2) Dairy farms located in provinces of Jeonbuk (A, B), Jeonnam (D, F, J), Gyeongnam (E, G), Chungnam (C), Gyeonggi (H), and Gangwon (I). The cheese types from farms A, E, G, H and J, Gouda; while B, C, D, I, Cheddar and Gouda; and F, Cheddar.
