# Supplementary material for: Characterisation of fungal contamination sources for use in quality management of cheese production farms in Korea
Source: Asian-Australas J Anim Sci. 2019 Oct 21;33(6):1002–11. doi: 10.5713/ajas.19.0553 (PMC7206383; doi:10.5713/ajas.19.0553)
Supplement: Supplementary file 1 [file ajas-19-0553-suppl.pdf]

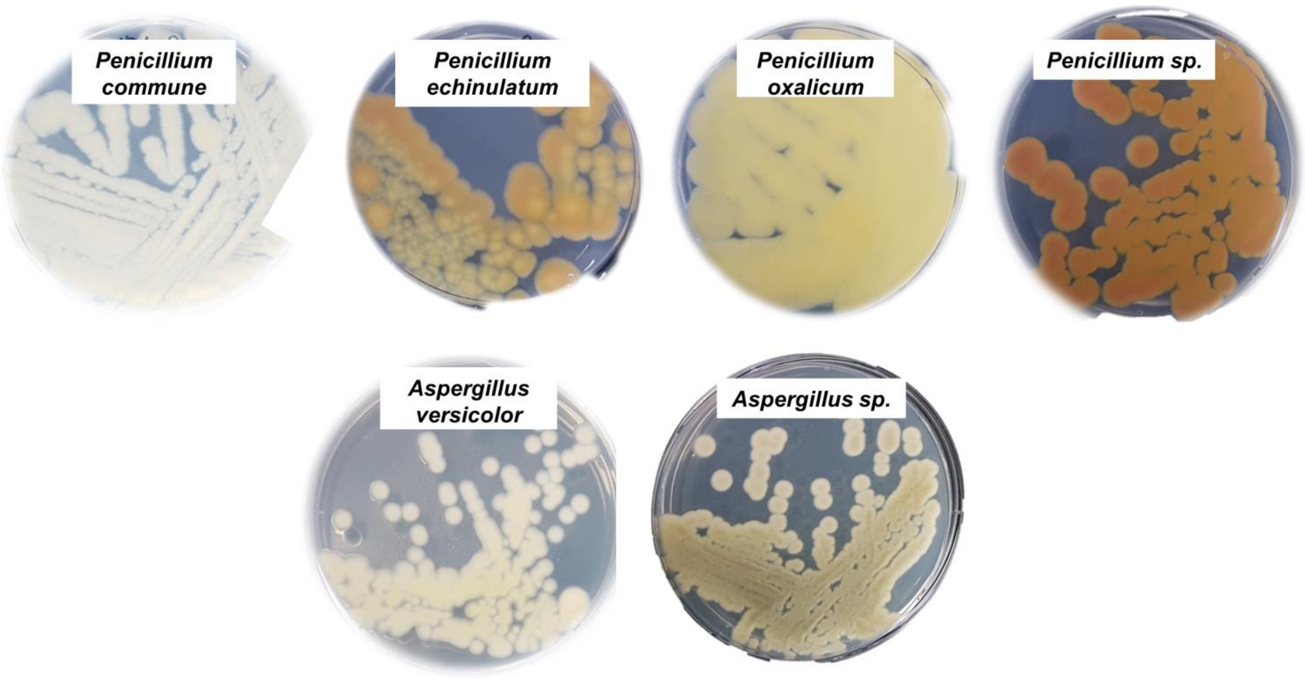

Supplementary Figure 1. Colony morphology of the predominant fungal species isolated in dairy farms
